# Clinical and Psychosocial Impact of Communication about Oral Potentially Malignant Disorders: A Scoping Review

**DOI:** 10.3390/dj11090209

**Published:** 2023-09-04

**Authors:** Lady P. A. Arboleda, Thaís C. E. Pereira, Joel B. Epstein, Cesar A. Migliorati, Saman Warnakulasuriya, Márcio Diniz-Freitas, Marcio A. Lopes, Alan R. Santos-Silva

**Affiliations:** 1 Oral Diagnosis Department, Piracicaba Dental School, University of Campinas, Piracicaba 13414-903, SP, Brazil; paola9228a@gmail.com (L.P.A.A.); thaiscep@gmail.com (T.C.E.P.); malopes@fop.unicamp.br (M.A.L.); 2 Graduate Program, A.C. Camargo Cancer Center, São Paulo 01508-020, SP, Brazil; 3 Samuel Oschin Comprehensive Cancer Center, Cedars-Sinai Health System, Los Angeles, CA 90048, USA; jepstein@coh.org; 4 Division of Head and Neck Surgery, City of Hope National Medical Center, Duarte, CA 91010, USA; 5 Department of Oral & Maxillofacial Diagnostic Sciences, College of Dentistry, University of Florida, Gainesville, FL 32603, USA; c.migliorati@dental.ufl.edu; 6 WHO Collaborating Centre for Oral Cancer and Faculty of Dentistry, Oral and Craniofacial Sciences, King’s College London, Londos WC2R 2LS, UK; s.warne@kcl.ac.uk; 7 Medical-Surgical Dentistry Research Group (OMEQUI), Health Research Institute of Santiago de Compostela (IDIS), University of Santiago de Compostela (USC), 15782 Santiago de Compostela, Spain; marcio.diniz@usc.es

**Keywords:** oral potentially malignant disorders, communication, truth-telling, scoping review

## Abstract

Delivering bad news has been widely studied in cancer, thus, this scoping review aims to identify the available evidence concerning the communication of oral potentially malignant disorders (OPMDs) and their clinical and psychosocial impacts. A search was performed using electronic databases (Medline/PubMed, Scopus, Embase, and Web of Science) and one grey literature database (Google Scholar). Studies focused on communicating the diagnosis of OPMDs and the patients’ perceptions were included. Study selection and data extraction were performed by two authors in a two-phase process. Five publications were included in the qualitative analysis. Differences regarding the study design, population, OPMDs assessed, and outcomes of professional–patient communication were found in each study. Protocols for OPMD communication have not yet been reported and there is a need to standardize strategies as communication skills may provide better clinical outcomes for patients diagnosed with potentially malignant disorders. Although future studies are needed, a brief list recommending the aspects that must be communicated is proposed.

## 1. Introduction

Oral potentially malignant disorders (OPMDs) are a group of conditions that predispose oral mucosa to malignant transformation, specifically to oral squamous cell carcinoma (OSCC), the most common head and neck cancer in adults. Although the minority of these disorders progress to cancer, early diagnosis is particularly important given the high mortality rate of late-stage OSCC [1].

It has been estimated that the overall worldwide prevalence of OPMDs is around 4.5%, with wide differences according to geographic region [2]. Although the overall malignant transformation rate across all OPMD groups is relatively low (7.9%), and each type of disorder has a highly variable rate of transformation (ranging from 1.4% to 49.5%), the risk of progression to OSCC is always a possibility and should be considered in the clinical follow-up of all patients affected by OPMDs [3].

The OPMDs present heterogeneous etiologies and their biology is characterized by mutations in the genetic codes of oral epithelial cells with or without clinical and histomorphological alterations that may lead to OSCC development [4]. According to the World Health Organization Collaborating Centre for Oral Cancer (2020), the OPMD group is composed of: leukoplakia, proliferative verrucous leukoplakia (PVL), erythroplakia, oral submucous fibrosis (OSF), oral lichen planus (OLP), actinic keratosis (actinic cheilitis) (AK/AC), palatal lesions in reverse smokers, oral lupus erythematosus (OLE), dyskeratosis congenita (DC), oral lichenoid lesion (OLL), and oral graft versus host disease (OGVHD) [1].

Communication with the patient has been recognized as one of the most important skills used by practitioners to help approach difficult issues and focus on patients’ values and preferences. Professional–patient communication has several potential positive outcomes, including reduced patient anxiety, increased patient satisfaction, motivation and adherence to healthy behaviors, and better oral health outcomes [5,6,7]. Delivering bad news has been widely studied in oncological settings, however, communication protocols for the diagnosis of OPMDs are unknown, even with knowing the clinical and psychosocial impact. Thus, this review seeks the relevant and sensitive aspects of communication following the diagnosis of an OPMD, emphasizing topics such as the risk of malignant transformation, signs and symptoms observed, changes in lifestyle, cessation of exposure to risk factors, uncertainties related to treatment, and the necessity for lifelong follow-up [8,9,10,11,12,13].

Since professional–patient communication about the diagnosis of OPMDs has been sparsely addressed in the scientific literature, a scoping review was the preferred study design by the authors, rather than a systematic review, to examine a comprehensive range of available sources and synthesize evidence on communication techniques, truth-telling in OPMDs communication, and the clinical and psychosocial impacts on patients. Moreover, we intend to report the gaps in the knowledge for future primary studies that investigate communication strategies for patients diagnosed with OPMDs.

## 2. Materials and Methods

### 2.1. Protocol and Registration

This scoping review was performed according to the Preferred Reporting Items for Systematic Reviews and Meta-Analyses extension for Scoping Reviews (PRISMA-ScR) [14] (Supplementary File S1). A protocol describing the research design was registered on Open Science Framework (OSF) (https://osf.io/az3fy, accessed on 31 August 2021).

### 2.2. Information Sources and Search

Medline/PubMed, Embase, Web of Science, and Scopus databases were searched for studies published before 12 October 2021. Additionally, a search on the grey literature (Google Scholar) was carried out and the reference lists of included studies were manually screened looking for additional relevant studies. The search was conducted by combining three groups of keywords (communication, oral potentially malignant disorders, and oral cavity), each of them containing their synonyms or related keywords and combined with the Boolean operator “and”. Supplementary Table S1 shows the search strategy used in each database.

### 2.3. Selection of Sources of Evidence

Once the search was completed, all citations were uploaded into EndNote X7 (Clarivate Analytics, Philadelphia, PA, USA) and duplicate records were removed. The titles and abstracts of all studies identified in the electronic searches were individually read by two reviewers (L.P.A.A. and A.R.S.S.), excluding articles that clearly did not meet the eligibility criteria using the online software Rayyan (Qatar Computing Research Institute, Doha, Qatar, https://www.rayyan.ai/) [15]. The two reviewers proceeded with reading the full text of the articles screened to identify the eligible articles and all the primary reasons for exclusions were registered. The study selection was always based on the full-text assessment.

The inclusion criteria of this scoping review were applied following the questions based on the PCC (population, concept, and context): Are there protocols for correctly informing diagnosis patients with OPMDs? When correctly informed, what is its clinical and psychosocial impact? Including, population: patients diagnosed with OPMD with no restrictions regarding sex, ethnicity, age, or geographic location. Concept: studies related to the main topics that a patient with OPMD should be aware of (clinical manifestations, the probability of progressing to OSCC, risk factors of OPMDs, treatment uncertainties, lifelong follow-up, and psychosocial impacts). Context: studies describing communication strategies, recommendations, or protocols, with an emphasis on the perception of patients and clinicians about the diagnosis and management of OPMDs. No restrictions regarding language or publication date were applied.

The following exclusion criteria were applied: (1) studies of oral conditions other than OPMD; (2) potentially malignant conditions in anatomical sites other than the oral cavity; (3) clinical trials focused only on screening, risk factors, diagnosis or diagnostic test accuracy, and treatment of OPMDs; (4) laboratory research with animal experimentation and in vitro studies, conference abstracts, posters, book chapters, and studies with the full-text not available; and (5) overlapping information; we included the most recently reported or those providing more data.

### 2.4. Data Synthesis and Descriptive Analysis

From the included studies, a data sheet was created for the extraction of data regarding the publication characteristics (authors, study design, country, and publication year), OPMD type, and communication characteristics according to the relevant topics for clinicians and patients when an OPMD is diagnosed. Due to the strong evidence gaps that were noted about communication of bad news on OPMDs, the authors designed descriptive recommendations related to the main topics that the health professional should be aware of when communicating to the patient with an OPMD, considering the patient’s preferences and values. These recommendation strategies were built by carefully analyzing all the aspects and topics addressed in the different published outcomes related to the professional perception of OPMD diagnosis.

## 3. Results

### 3.1. Selection and Characteristics of Sources of Evidence

**Figure 1:**
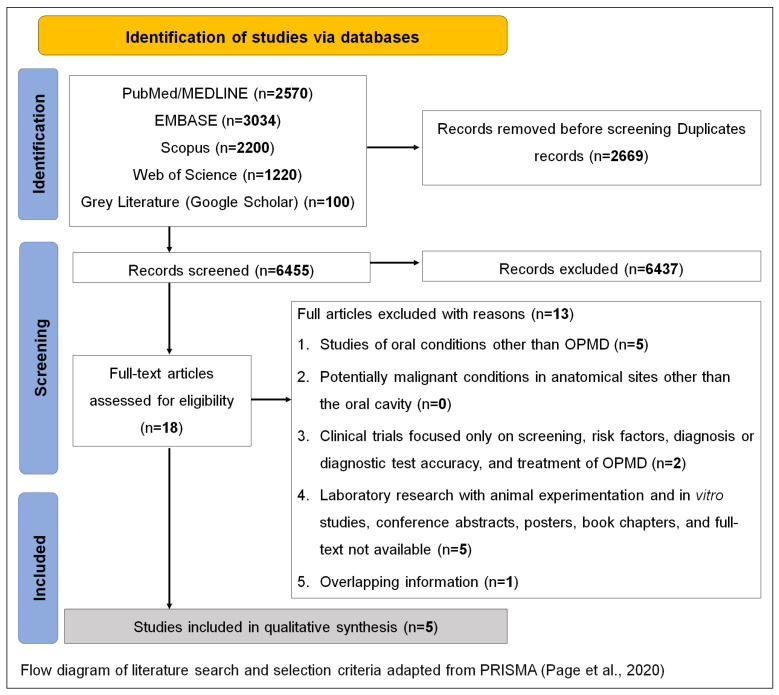
Flow diagram of literature search and selection criteria adapted from PRISMA [21].

The search resulted in 9124 identified records; 6455 records remained after duplicates were removed. A total of 6437 records were excluded during the initial screening of titles and abstracts, with 18 studies remaining for phase 2 of the study selection. After a full-text assessment, 13 studies were excluded (Supplementary Table S2) and 5 studies were included in the scoping review, of which 1 was a comment [16], 1 was a review [17], 2 were qualitative studies [18,19], and 1 reported a case series [20] (Figure 1).

Two studies assessed oral leukoplakia exclusively, two others evaluated OPMDs without describing which clinical subtypes were included, and one study reported 13 cases of OPMDs, including oral leukoplakia, palatal lesions in reverse smokers, erythroplakia, PVL, OLP, OLL, OLE, and OSF. The United Kingdom (*n* = 1), the Netherlands (*n* = 2), India (*n* = 1), and Taiwan (*n* = 1) were the countries where the included studies were carried out. Table 1 summarizes the characteristics of the five selected studies.

### 3.2. Synthesis of Results

The results related to the communication themes that were part of the purpose of this scoping review are presented in Table 2. We could identify some critical issues regarding OPMD communication, such as insecurity in talking about the diagnosis, the need for training on communication techniques, and inadequate patient health literacy [16,18,19]. According to the main topics covered in the literature, communication on OPMDs related to risk factors, malignant transformation, treatment approaches, follow-up approaches, and clinical/psychosocial impacts were collected. In addition, patients’ preferences and some general recommendations reported in the included studies were obtained. Therefore, the absence of specific protocols on how to communicate the diagnosis of OPMDs creates a problem as it is necessary to identify information relevant to the patient and tell the truth when communicating about OPMDs. We created a list of recommended strategies for OPMD communication as shown in Table 3.

## 4. Discussion

We reviewed studies from different parts of the world, noting the clinical and psychological impacts that giving bad news relaying an OPMD diagnosis has on patients and their families. Unfortunately, there are no studies focused on communication protocols for patients who are diagnosed with OPMDs. For this reason, the present scoping review extracted and synthesized the main results that are relevant to OPMD communication. We mainly focused on the following aspects that we consider imperative when diagnosing an OPMD: risk factors related to the disorder, malignant transformation rates, physical impairment and functional limitations, psychological and social impacts, and treatment-related details (treatment uncertainties, effects of treatment on daily life, and lifelong follow-up).

### 4.1. Challenges for Professionals in Delivering Bad News Regarding OPMDs

There are protocols based on communicating bad news in the medical context and, in relation to the dentistry field, a recent review of the communication protocols in oral cancer patients showed available models such as SPIKES and ABCDE which recommend communication techniques considering patients’ preferences [7,22]. In a personal-view study on telling the truth to patients with cancer, the author highlighted the following which could also be applicable in the context of OPMDs: “when the relationship between patients and their oncologists is recognized as an open-ended dynamic process of ascertainment and constant reassessment of a truth shared between them, it acquires a different strength and character. Truth-telling then becomes a bidirectional process aimed at constructing—rather than merely discovering—the truth and at helping people with cancer to make sense of having and living with their disease” [6].

OPMD communication carries several challenging points for professionals, as there is still controversy about the different diagnostic techniques, the correlation with the histopathological characteristics, the uncertainties with the choice of treatment, and the probability of disease recurrence or turning into cancer, among others. All this means that the scientific evidence has not yet reached consensus or uniformity with the different techniques of diagnosis, treatment, and follow-up [16,17].

Health literacy has been reported as one of the most important factors to take into consideration when communicating bad news and represents a challenge for health professionals, as several studies demonstrate the difficulty of communication with patients possessing inadequate health literacy [9,23]. In oncology, for example, one study showed that adequate health literacy is necessary in terms of understanding and using cancer prevention and early detection strategies. In addition, patients are unaware of the main symptoms and signs of cancer, which may lead to a late diagnosis. On the other hand, there are verbal and written communication barriers that generate difficulties in relation to cancer treatment, as there are risks and benefits that must be understood and communicated correctly prior to decision-making [24]. The aforementioned challenge shows an interesting point that we must take into account when communicating about an OPMD since knowing the patient’s health literacy level can help with the necessary tools, as well as the appropriate words, to deliver the OPMD diagnosis.

### 4.2. Communication about Risk Factors Related to OPMDs

There is a group of known risk factors associated with OPMDs such as tobacco use, alcohol consumption, betel quid chewing, sun exposure, and, to a lesser extent, the transmitted infection of human papillomavirus (HPV, mainly type 16) and oral microbiome alteration, among others, that are well recognized [25]. Communication on the risk factors was shown in one study that reported proactivity by dentists in talking about smoking cessation, however, some of the professionals were not comfortable talking about alcohol as a risk factor or quitting/the moderation of alcohol use [18]. Communication on risk factors directly depends on the geographic region and the prevalence of OPMD as certain cultural risk factors influence the type and pattern of disorders. For example, betel quid/areca nut chewing habits are widely prevalent in South Asian populations, resulting in a greater prevalence of OPMDs [26]. Another challenging component in risk factor communication is when an OPMD is found in patients with different epidemiological profiles and with no exposure to an environmental factor, in other words, factors other than tobacco and alcohol may be implicated in the development of oral cancer as encountered in some younger patients. The dentist must be able to provide a balanced biological context for patients’ questions about their OPMD diagnosis and the absence of external risk factors as well when they are working with people exposed to risk factors without a diagnosis of OPMD.

### 4.3. Communication about Rates of Malignant Transformation

Reporting rates of malignant transformation must be within the epidemiological and clinicopathological context of each patient, as each type of OPMD has a highly variable rate of malignant transformation [3]. Currently, the grade of dysplasia present within an OPMD is seen as the most reliable marker for malignant transformation [27]. However, investigations on the molecular techniques used for assessing the prognostic value of biomarkers for OPMD are still insufficient to support malignant transformation, especially regarding their clinical application [4]. In this scoping review, it was not possible to observe the direct results of patient communication on the rates of malignant transformation, however, we found some studies that reported higher degrees of anxiety when the patient was informed about the chance of the lesion progressing into cancer [19,28].

### 4.4. Treatment-Related Communication

Treatment-related communication in the OPMD context is also complicated by a lack of robust evidence concerning both the treatment effectiveness for OPMDs and future OSCC risk [29]. The decisions related to the type of treatment are the most controversial in the literature since this decision should be based on the published evidence, circumstances, and context of each patient. It is necessary to inform the patient about the uncertainties in the outcomes of treatment and always lay out the facts so they do not feel disappointed when having to repeat the same intervention or change the direction of management [9]. Follow-up protocols change depending on the type of OPMD. Furthermore, there is no consensus on the specific time interval for follow-up/surveillance as there are no studies showing efficacy regarding better clinical outcomes [16,17]. However, periodic follow-up visits are advised in all OPMD cases [30]. Patients must understand that, although the time interval depends on clinical criteria, they will need to undergo lifelong follow-up.

### 4.5. Communicating Clinical/Psychosocial Implications to Patients

No specific protocol studies were found to learn about communicating the clinical and psychological impacts on patients who are diagnosed with OPMD. However, during the literature search carried out in this study, we observed that there are many studies concerning the quality of life of patients diagnosed with OPMDs, particularly, those related to lichen planus, leukoplakia, and oral submucous fibrosis [8,9,10,11,12,13]. The findings of these studies suggested that, in general, the signs and symptoms generated by OPMDs are the most important factors due to physical impairment and functional limitations. OPMD has a debilitating effect on psychological well-being and social interactions, thus, patients should be informed about future physical and psychosocial problems and try to delineate treatment plans focused on reducing these impacts.

### 4.6. Patients’ Preferences on OPMD Communication

The patient’s perception of the OPMD diagnostic process has been reported in screening studies and diagnostic test accuracies that reported patients’ values and preferences in the assessment of clinically evident lesions in the oral cavity [30,31]. The three main topics reported by the authors were: (i) fear and anxiety as some of the most relevant barriers to seeking care; (ii) the acceptability of conducting a clinical examination to identify OPMD; and the last and most important: (iii) participants highlighting an interest in being educated about ways to reduce their risk of having oral cancer and suggesting that mass media coverage could be an effective way to increase awareness about the early manifestation of OPMD and OSCC. Nevertheless, the authors conclude that more information on patients’ values and preferences is required [30]. Studies on web-based information have revealed the presence of misinformation in the electronic media on the subject of OPMDs and the necessity to develop and portray accurate information on this topic to the general public [32,33]. Professional organizations concerned with oral medicine have a duty to publish such electronic patient information leaflets.

### 4.7. General Recommendations on OPMD Communication

The diagnosis of OPMD can occur in private practice by a clinician or at academic institutions. Thus, communication skills are recommended as part of the curriculum in dental schools [34]. Breaking bad news might not only be challenging for the patient and caregivers but also for a student without any experience [35]. Worked examples and simulated patients are resourceful strategies that could help with teaching these difficult communication skills to students [35,36,37]. Communication skill training could also include role-play sessions, videos on patient communications, presentations, and experience sharing from tutors and senior students [38].

Our findings clearly indicate that more qualitative investigations are needed to determine communication protocols for each type of OPMD as well as to identify the perception of professionals and patients. As noted, only leukoplakia was directly related to communication, and its author outlined relevant information on how this information should be reported to a patient [16,17]. Therefore, it is necessary to implement adequate communication strategies and to provide effective communication protocols for a full range of OPMDs.

The potential limitation of the present scoping review was the limited data reported on answering the communication protocols of OPMD diagnosis. Future studies should focus on determining what information is provided for patients diagnosed with common OPMDs and, on the other hand, determining what questions patients have asked their dentists and what information they prioritize about OPMDs. The main strength of our study is its originality, as it is the first scoping review that attempts to address the main highlights of OPMD communication based on scientific evidence.

## 5. Conclusions

Finally, the most obvious finding to emerge from this study is that there are no communication protocols for patients who are diagnosed with OPMDs. Healthcare professionals must develop and practice communication skills throughout their training and practice, starting by incorporating specific training in the dental school curriculum. Due to the limitation in the time available in clinical settings, developing and making available an easily accessible and accurate web-based patient information sheet that could be recommended to an OPMD patient should be considered by professional bodies. Recommendations such as applying the SPIKES protocol in clinical practice and telling the truth to the patient, based on scientific evidence, are strategies exposed in this scoping review.

## Figures and Tables

**Table 1:** Characteristics of the five included studies.

#	Author (Year)	Country	Study Design	Population	Sample	OPMD Studied	Thematic Aspects of OPMD Communication
1	Brocklehurst et al. (2010) [18]	The United Kingdom	Semi-structured interviews	Dental practices	18	OPMD	Information that is given to the patient. The patient’s response to being told that a potentially malignant lesion has been detected. The advice given to the patient about the known risk factors of the malignant disease. Comments on the management of potentially malignant disorders in practice before a referral is made. Practical aspects of the referral process detailing how dentists refer and who they send their referrals to.
2	Raman P. (2021) [20]	India	Case report	Patients with OPMD	13	Oral leukoplakia Palatal lesions in reverse smokers erythroplakia PVL OLP OLL OLE OSF	Communication and habit counseling of patients with OPMD.
3	Van der Waal I. (2018) [16]	The Netherlands	Comment	NA	NA	Leukoplakia	This study discussed how the subject of oral leukoplakia might be communicated among various healthcare workers and among patients. The article comments on aspects such as definition, clinical classification, biopsy, and how the presence of epithelial dysplasia is an important risk marker of malignant transformation.
4	Van der Waal, I. (2019) [17]	The Netherlands	Review	NA	NA	Leukoplakia	How to inform a patient who has a leukoplakia.
5	Lin H, et al. (2015) [19]	Taiwan	Cross-sectional descriptive study	Patients with OPMD	106	OPMD	This study investigated: anxiety, attitudes towards cancer prevention, and unmet information needs; differences in anxiety and attitudes towards cancer prevention between met and unmet information needs; and the associated factors of unmet information needs for patients with precancerous oral lesions.

Legend: OSF: oral submucous fibrosis; OLP: oral lichen planus; OLE: oral lupus erythematosus; OLL: oral lichenoid lesion; and PVL: proliferative verrucous leukoplakia.

**Table 2:** Findings according to OPMD communication.

OPMD Themes	Findings
Insecurity with talking about the diagnosis	“The most common explanation given to patients, once a lesion was found, was that the dentist would like a second opinion” [18]. “There was a concern expressed by some primary care dentists about the problem of not providing the patient with enough information to prompt them to attend for their appointment with secondary care” [18].
Need for training on communication techniques	“The appropriate use of language was a concern for many dentists in order to avoid patient anxiety. In fact, some would deliberately describe the lesion in different terms, avoiding any terms associated with malignancy” [18]. “The present study suggests that more can be done to train primary care dentists in health promotion and patient communication” [18].
Patient health literacy	“It is a challenge to properly inform patients affected by leukoplakia. Some patients are very well educated and are looking for rather detailed information at a high, sometimes even academic level. They may want to be involved in the decisions that have to be taken, for example, in the taking of a biopsy or, even more so, in the decision to be treated or not to be treated” [16]. “The majority of patients, want to be guided in their decision-making by their doctor and will ask for clear and concise information. One should realize that most of the available information on oral leukoplakia, in writing or through the Internet, is much too complicated for lay people” [16]. “Health education and individual counselling should be provided to satisfy the information needs of this population” [19]. “Patients with unmet information needs had higher levels of anxiety than those whose information needs were met. Poor health literacy for patients who had betel nut use, low health literacy and insufficient skills for obtaining, reading and understanding information to make appropriate health decisions” [19].
Risk factors	“While some of the dentists did provide leaflets at their practice, there were also some who did not believe in providing patients with any more information” [18]. “Many practices were proactive in talking about smoking cessation” [18]. “A common complaint among those dentists who had tried to provide smoking cessation was that they felt frustrated because it had no unit of dental activity value and they could not prescribe the nicotine replacement therapy” [18]. “Further work is required to understand why dentists do not feel comfortable talking about alcohol as a risk factor” [18]. “Patients with precancerous oral lesions who had high levels of state anxiety, long duration of time since quitting betel nut chewing and were without a history of oral cancer were more likely to have unmet information needs” [19]. “The participants in our study reported betel nut use and showed passive motivation for regular oral mucosal screening, indicating that they were at risk for developing pre-malignant oral lesions” [19]. “Enhancing provider–patient interaction and presenting essential information first can help patients follow the instructions for cancer prevention” [19]. “Unmet information needs were associated with the time since quitting betel nut chewing and a history of oral cavity cancer. Patients who had quit using a harmful substance and who also had previous illness experiences were different from those who were willing to adopt health promotion behavior such as cancer prevention. Because of their prior experience of illness and their decision to quit betel nut chewing, these patients might have a higher intention to participate in oral mucosal screening, including regular follow-up testing and future cancer prevention program” [19].
Malignant transformation	Leukoplakia: “A probably frequently occurring confusion is that, in the absence of epithelial dysplasia, the pathologist may conclude his report by saying “This is not a premalignant lesion.” As mentioned before, oral leukoplakia is primarily a clinical term without specific histopathological features. At histopathological examination, one may or may not observe epithelial dysplasia” [16]. “The patient should be informed that the leukoplakia may recur within a period varying from some weeks, months, or several years. They also should know that the risk of oral cancer development may not be eliminated by the excision. Although the efficacy of follow-up visits has never been shown, it seems preferable to offer such visits, mainly for reassurance of the patient” [17].
Treatment approaches	“Oral leukoplakia can be treated by a variety of modalities such as cold-knife surgery or laser surgery, CO_2_ evaporation, photodynamic treatment, and non-medical treatments. As has been shown in numerous studies, including a Cochrane review, not any of these treatment modalities are truly effective in preventing or decreasing the risk of malignant transformation. Therefore, the question remains whether or not to treat oral leukoplakia” [16]. “Spontaneous regression of non-dysplastic leukoplakia is in my experience extremely rare as well” [16]. “The increased morbidity in such instances should be properly weighted against the expected benefit of the treatment” [16]. “In large, diffuse or multiple oral leukoplakias, one may choose to perform an “excisional” biopsy of the clinically most suspected area only, if present, or to perform multiple biopsies (mapping). In any case, the patient should play an important role in this shared decision taking. Some will prefer not to have active treatment while others persist to be treated, even in case of extensive or multiple oral leukoplakias. A similar divergence in opinion may arise in case of recurrence. Some patients do not want to undergo treatment again, while others insist on retreatment” [17]. “The inadequate expression of emotions and lack of stress release may interfere with information-seeking and treatment decisions. Support and listening are needed to help these patients deal with the treatment-decision process” [19].
Follow-up approaches	“There is no evidence that lifelong follow-up programs for treated or untreated patients with leukoplakia are effective in preventing the development of oral cancer. Most likely, follow-up programs will not result in improved survival in case of cancer development either. Nevertheless, it is common practice, i.e., For reassurance of the patient, to follow up the patients. Depending on various aspects, such as the extent of the leukoplakia and the presence and degree of epithelial dysplasia, intervals may vary from 3 to 6 months, lifelong. Changes in the clinical presentation and, particularly, symptoms are ominous signs of malignant transformation” [16]. “It is well understood that such follow-up programs may not be feasible all over the world. Besides, there is the issue of patients’ compliance. After several years of uneventful follow-up, some patients will discontinue the follow-up program” [16].
Clinical/psychosocial impacts	“Patients reported their mouth condition having a debilitating effect on their psychological well-being and social interactions” [9]. “Physical impairment and functional limitations’ were the most important theme for many of the patients” [9]. “The impacts of OPMD also extended beyond physical impairment and functional limitations to aspects of daily living, notably psychological and social wellbeing” [9]. “A high level of anxiety about precancerous oral lesions was more prevalent among patients with unmet information needs than among those whose information needs were met” [19].
Patient preferences on OPMD communication	“The majority of the dentists questioned suggested that patients were not overly distressed about a positive screen, they just go along with the suggestion” [18]. “Most patients will not be interested to listen to an academic lecture by their doctor on the various aspects of oral leukoplakia but, instead, they want to be informed in an understandable way particularly when it comes to the further management” [17]. “Patients reported higher information needs related to ‘To be fully informed about your test results as soon as possible.’ and ‘To be fully informed about all of the benefits and side effects of treatment or surgery before you agree to have it.” [19].
Recommendations	“A primary care physician should be responsible, humble, knowledgeable, and skillful to deliver an effective holistic care by inculcating the practice of effective communication of bad news, timely habit cessation counseling and compassionate care as a part of routine dental screening” [20]. “There is a delayed presentation of oral pre cancer and oral cancer in India, as approximately 50% of patients are diagnosed at last stage since the asymptomatic pre cancer lesions are missed by oral physicians/dentists either due to lack of timely communication and habit counseling, lack of knowledge, or inappropriate attitude, putting all in a nut shell—sheer lack of empathy and commitment towards patient care and society” [20]. “The author believes that the three most important, least explored and challenging palliative care approaches namely, “Communication,” “Counseling,” and “Compassionate care,” should be effectively practiced by a primary care physician, to improve their level of commitment to society and attitude towards patient care which can help in early diagnosis of OPMD and decreased incidence of oral cancer, thus improving quality of life of patients” [20]. “Communication on this subject with patients should be in easy to understand wording, avoiding professional terminology as much as possible” [16]. “One may consider to send a brief summary of the discussion held with the patient in easy to understand language” [16].

**Table 3:** Recommendation strategies on patient information in OPMD communication according to the problems and needs observed in the literature.

OPMD Themes	Findings
Communication technique SPIKES protocol [12,22]	S: Setting ∘ Prepare for the invitation by reviewing the notes and inviting the patient to involve people important to them. ∘ Prepare the environment, ensure sufficient time is available for consultation and privacy. ∘ Take note of body language, be seated, not standing. P: Perception ∘ Find out the patient's perception of their illness. I: Invitation ∘ Find out how much information they would like, and to what level of detail. K: Knowledge ∘ Impart the bad news clearly and simply, avoiding jargon, with frequent pauses to check for understanding. ∘ Use a ‘warning shot’ statement first so that patients are prepared that bad news is coming. E: Empathy ∘ Allow the patient to express their emotions, using empathic responses to acknowledge their feelings and show support. S: Summarize and strategize ∘ Make a plan with the patient for the future and summarize the discussion; check for the patient’s understanding.
Telling the truth about: risk factors, malignant transformation, treatment approaches, follow-up approaches, and clinical/psychosocial impacts	Know your patient’s health literacy level to define the methodology and communication tools that will be used to inform the OPMD diagnosis. Have leaflets with images that help explain the diagnosis to the patient. Avoid professional terminology as much as possible. Speak in proper terms about the malignancy “the white or red patch can turn into cancer”. Inform about the risk factors associated with diagnosed OPMD and explain the scientific reasons for this association. Raise awareness of the importance of avoiding lifestyle risk factors when they are present. Inform about the potential malignancy rate of diagnosed OPMD according to the clinical, demographic, and geographic characteristics of the population in which the patient is inserted. Talk about the uncertainties that exist in determining whether the diagnosed OPMD will change into oral cancer. Inform about the main clinical manifestations of diagnosed OPMD as well as the impact that these manifestations could have on daily life. Explain the available treatment modalities and make a decision prioritizing the patients’ well-being, the potential morbidities of treatment (e.g., excision of large areas), and their values and preferences. Talk about the uncertainties that exist regarding recurrence and malignant transformation, even after treatment. Raise awareness of the need for continuous follow-up throughout life, especially with the aim of avoiding late diagnosis. Explain that the interval between follow-up appointments will depend on several factors, such as clinical characteristics, the professional’s judgment, and updates to the scientific evidence on the follow-up protocols for each OPMD. Although a patient’s health literacy is relevant to understanding their condition, ask the patient repeatedly about their doubts and emphasize the most important points until he/she fully understands. Explain the need to observe any changes in symptoms (e.g., pain) and report back even before the next review appointment.
Recommendations for dental students	In the academic setting, dental students must repeatedly accompany the senior professional in communicating bad news in order to have the opportunity to learn and practice before carrying out the communication alone. Training in communication skills, the SPIKES protocol, and possible emotional reactions from patients and caregivers. The dental school curriculum should also include the knowledge to answer the questions the patients and caregivers might have. Different teaching modalities by means of education and practice are recommended, such as worked examples and simulated patients, role-play sessions, videos on patient communications, presentations, and experience sharing from tutors and senior students.

## Data Availability

Data availability are available on the Open Science Framework (https://doi.org/10.17605/OSF.IO/AZ3FY). If additional data is needed, the authors would make it available according to reasonable requests.

## Supplementary Materials

The following supporting information can be downloaded at: https://www.mdpi.com/article/10.3390/dj11090209/s1, Table S1: Database search strategy; Table S2: Excluded articles and the reasons for exclusion (*n* = 13); File S1: PRISMA-ScR checklist.
