# Supplementary material for: Clinical and Psychosocial Impact of Communication about Oral Potentially Malignant Disorders: A Scoping Review
Source: Dent J (Basel). 2023 Sep 4;11(9):209. doi: 10.3390/dj11090209 (PMC10530221; doi:10.3390/dj11090209)
Supplement: Supplementary file 1 [file dentistry-11-00209-s001.zip › Supplementary Tables.pdf]

Supplementary Material

Supplementary Table S1. Database search strategy

Database	Search strategy<br>(search date: October 12, 2021)	Results
**MEDLINE/PubMed**	(“Truth Disclosure"[Mesh] OR “Truth Disclosure" OR "Truth-telling" OR "Truth telling" OR "Telling the Truth" OR Truth OR Truthing OR Truths OR "Access to Information" OR "Professional-Patient Relations" OR "Health Planning Guidelines"[Mesh] OR "Health Planning Guidelines" OR "Health Planning" OR Guideline OR Guidelines OR "Planning Guideline" OR "Planning Guidelines" OR "Guidelines for Health Planning" OR Recommendations OR Recommendation OR "Health Planning Recommendations" OR "Planning Recommendations" OR "Communication"[Mesh] OR Communication OR Communications OR Misinformation OR "Teach-Back Communication"[Mesh] OR "Teach-Back Communication" OR "Closed-Loop Communication" OR "Closed Loop Communication" OR "Closed-Loop Communications" OR “Teach-Back” OR "Closed-Loop" OR "Health Communication"[Mesh] OR "Health Communication" "Health Communications" OR "Communication Barriers"[Mesh] OR "Communication Barriers" OR "Communication Barrier" OR "Dentist-Patient Relations"[Mesh] OR "Dentist-Patient Relations" OR "Dentist-Patient Relation" OR "Dentist Patient Relation" OR "Dentist-Patient Relationship" OR "Dentist Patient Relationship" OR "Dentist-Patient Relationships" OR "Dentist Patient Relationships" OR "Physician-Patient Relations"[Mesh] OR "Physician-Patient Relations" OR "Physician-Patient Relation" OR "Physician Patient Relation" OR "Physician Patient Relationship" OR "Physician Patient Relationships" OR "Physician Patient Relations" OR "Physician Patient Relations" OR "Doctor Patient Relations" OR "Doctor Patient Relation" OR "Doctor-Patient Relations" OR "Doctor-Patient Relation" OR "Delivering bad news" OR "bad news" OR "receiving bad news" OR "deliver bad news" OR "communicate bad news" OR "Communication of bad news" OR "Breaking bad news" OR "break bad news" OR Protocol OR Protocols OR "Clinical Protocols"[Mesh] OR "Clinical Protocols" OR “Clinical Protocol” OR Questionnaires OR Questionnaire OR "Quality of Life"[Mesh] OR "Quality of Life") **AND** ("Precancerous conditions"[Mesh] OR precancerous OR "pre-cancer" OR precancer OR preneoplastic OR "pre-neoplastic" OR "precursor lesion" OR "precursor lesions" OR "potentially malignant disorder" OR "potentially malignant disorders" OR "potentially malignant lesion" OR "potentially malignant lesions" OR premalignant OR "pre malignant" OR pre malignancy OR "pre malignancy" OR pre malignancies OR "pre malignancies" OR "Carcinoma in Situ"[Mesh] OR "Carcinoma in situ" OR "in situ carcinoma" OR "Carcinomas in situ" OR "in situ carcinomas" OR "preinvasive carcinoma" OR "preinvasive carcinomas" OR "pre-invasive carcinoma" OR "pre-invasive carcinomas" OR "intraepithelial carcinoma" OR "intraepithelial carcinomas" OR "intraepithelial neoplasm" OR "intraepithelial neoplasms" OR "intraepithelial neoplasia" OR "intraepithelial neoplasias" OR "intraepithelial cancer" OR "Leukoplakia, Oral"[Mesh] OR "Leukoplakia"[Mesh] OR leukoplakia OR leukoplakias OR leukoplakic OR keratosis OR keratoses OR leukokeratosis OR leukokeratosis OR "proliferative verrucous leukoplakia" OR "Erythroplasia"[Mesh] OR erythroplasia OR erythroplasias OR erythroplakia OR erythroleukoplakia OR "Oral Submucous Fibrosis"[Mesh] OR "Oral submucous fibrosis" OR "Submucous Fibrosis" OR "Lichen Planus, Oral"[Mesh] OR "Oral Lichen Planus" OR "Cheilitis"[Mesh] OR cheilitis OR "actinic cheilosis" OR "Actinic cheilitis" OR "Keratosis, Actinic"[Mesh] OR "Actinic Keratoses" OR "Actinic Keratosis" OR "Reverse-cigar smokers" OR "smoker's palate" OR "reverse smokers" OR "Oral Lupus Erythematosus" OR "Dyskeratosis Congenita"[Mesh] OR "Dyskeratosis Congenita" OR "Oral Lichenoid Lesions" OR "Oral Lichenoid Lesion" OR "Oral graft versus host disease" OR "oral graft-versus-host disease") **AND** ("Mouth"[Mesh Terms] OR "mouth" OR "mouths" OR "oral" OR "orals" OR "lip"[MeSH Terms] OR "lip" OR "lips" OR "tongue"[MeSH Terms] OR "tongue" OR "Mouth Mucosa"[MeSH Terms] OR "Mouth Mucosa" OR "buccal" OR "Palate"[MeSH Terms] OR "palate" OR "palates" OR "Mouth Floor"[MeSH Terms] OR "Mouth Floor" OR "cheek mucosa" OR "alveolar" OR "Gingiva"[MeSH Terms] OR "gingiva" OR "Gingivas" OR "gum" OR "gums" OR "interdental papilla" OR "interdental papillae" OR "commissure" OR "maxillary tuberosity" OR "uvula" OR "uvular")	**2,570**
**SCOPUS**	TITLE-ABS-KEY ("Truth Disclosure" OR "Truth-telling" OR "Truth telling" OR "Telling the Truth" OR truth OR truthing OR truths OR "Access to Information" OR "Professional-Patient Relations" OR "Health Planning Guidelines" OR "Health Planning" OR guideline OR guidelines OR "Planning Guideline" OR "Planning Guidelines" OR "Guidelines for Health Planning" OR recommendations OR recommendation OR "Health Planning Recommendations" OR "Planning Recommendations" OR communication OR communications OR misinformation OR "Teach-Back Communication" OR "Closed-Loop Communication" OR "Closed Loop Communication" OR "Closed-Loop Communications" OR "Teach-Back" OR "Closed-Loop" OR "Health Communication" OR "Health Communications" OR "Communication Barriers" OR "Communication Barrier" OR "Dentist-Patient Relations" OR "Dentist-Patient Relation" OR "Dentist Patient Relation" OR "Dentist-Patient Relationship" OR "Dentist Patient Relationship" OR "Dentist-Patient Relationships" OR "Dentist Patient Relationships" OR "Physician-Patient Relations" OR "Physician-Patient Relation" OR "Physician Patient Relation" OR "Physician Patient Relationship" OR "Physician Patient Relationships" OR "Physician Patient Relations" OR "Physician Patient Relations" OR "Doctor Patient Relations" OR "Doctor Patient Relation" OR "Doctor-Patient Relations" OR "Doctor-Patient Relation" OR "Delivering bad news" OR "bad news" OR "receiving bad news" OR "deliver bad news" OR "communicate bad news" OR "Communication of bad news" OR "Breaking bad news" OR "break bad news" OR protocol OR protocols OR "Clinical Protocols" OR "Clinical Protocol" OR questionnaires OR questionnaire OR "Quality of Life") AND TITLE-ABS-KEY ("Precancerous conditions" OR precancerous OR "pre-cancer" OR precancer OR preneoplastic OR "pre-neoplastic" OR "precursor lesion" OR "precursor lesions" OR "potentially malignant disorder" OR "potentially malignant disorders" OR "potentially malignant lesion" OR "potentially malignant lesions" OR premalignant OR "pre malignant" OR "pre malignancy" OR "pre malignancy" OR "pre malignancies" OR "pre malignancies" OR "Carcinoma in situ" OR "in situ carcinoma" OR "Carcinomas in situ" OR "in situ carcinomas" OR "preinvasive carcinoma" OR "preinvasive carcinomas" OR "pre-invasive carcinoma" OR "pre-invasive carcinomas" OR "intraepithelial carcinoma" OR "intraepithelial carcinomas" OR "intraepithelial neoplasm" OR "intraepithelial neoplasms" OR "intraepithelial neoplasia" OR "intraepithelial neoplasias" OR "intraepithelial cancer" OR leukoplakia OR leukoplakias OR leukoplakic OR keratosis OR keratoses OR leukokeratosis OR leukokeratosis OR "proliferative verrucous leukoplakia" OR erythroplasia OR erythroplasias OR erythroplakia OR erythroleukoplakia OR "Oral submucous fibrosis" OR "Submucous Fibrosis" OR "Oral Lichen Planus" OR cheilitis OR "actinic cheilosis" OR "Actinic cheilitis" OR "Actinic Keratoses" OR "Actinic Keratosis" OR "Reverse-cigar smokers" OR "smoker's palate" OR "reverse smokers" OR "Oral Lupus Erythematosus" OR "Dyskeratosis Congenita" OR "Oral Lichenoid Lesions" OR "Oral Lichenoid Lesion" OR "Oral graft versus host disease" OR "oral graft-versus-host disease") AND TITLE-ABS-KEY (mouth OR mouths OR oral OR orals OR lip OR lips OR tongue OR "Mouth Mucosa" OR buccal OR palate OR palates OR "Mouth Floor" OR "cheek mucosa" OR alveolar OR gingiva OR gingivas OR gum OR gums OR "interdental papilla" OR "interdental papillae" OR commissure OR "maxillary tuberosity" OR uvula OR uvular)	**2,200**
**EMBASE**	('truth disclosure'/de OR 'truth-telling' OR 'truth telling' OR 'telling the truth' OR 'truth'/de OR truthing OR truths OR 'access to information'/de OR 'professional-patient relations'/de OR 'health planning guidelines'/de OR 'health planning'/de OR 'guideline'/de OR 'guidelines'/de OR 'planning guideline' OR 'planning guidelines' OR 'guidelines for health planning' OR 'recommendations'/de OR recommendation OR 'health planning recommendations' OR 'planning recommendations' OR 'communication'/de OR communications OR 'misinformation'/de OR 'teach-back communication'/de OR 'closed-loop communication' OR 'closed loop communication' OR 'closed-loop communications' OR 'teach-back' OR 'closed-loop' OR 'health communication'/de OR 'health communications' OR 'communication barriers'/de OR 'communication barrier'/de OR 'dentist-patient relations'/de OR 'dentist-patient relation'/de OR 'dentist patient relation'/de OR 'dentist-patient relationship'/de OR 'dentist patient relationship'/de OR 'dentist-patient relationships' OR 'dentist patient relationships' OR 'physician-patient relations'/de OR 'physician-patient relation'/de OR 'physician patient relation'/de OR 'physician patient relationship'/de OR 'physician patient relationships' OR 'physician patient relations'/de OR 'doctor patient relations' OR 'doctor patient relation'/de OR 'doctor-patient relations' OR 'doctor-patient relation'/de OR 'delivering bad news' OR 'bad news' OR 'receiving bad news' OR 'deliver bad news' OR 'communicate bad news' OR 'communication of bad news' OR 'breaking bad news' OR 'break bad news' OR 'protocol'/de OR protocols OR 'clinical protocols'/de OR 'clinical protocol'/de OR 'questionnaires'/de OR 'questionnaire'/de OR 'quality of life'/de) AND ('precancerous conditions'/de OR precancerous OR 'pre-cancer'/de OR 'precancer'/de OR preneoplastic OR 'pre-neoplastic' OR 'precursor lesion' OR 'precursor lesions' OR 'potentially malignant disorder'/de OR 'potentially malignant disorders' OR 'potentially malignant lesion' OR 'potentially malignant lesions' OR premalignant OR 'pre malignant' OR 'pre malignancy' OR 'pre malignancies' OR 'carcinoma in situ'/de OR 'in situ carcinoma'/de OR 'carcinomas in situ' OR 'in situ carcinomas' OR 'preinvasive carcinoma'/de OR 'preinvasive carcinomas' OR 'pre-invasive carcinoma' OR 'pre-invasive carcinomas' OR 'intraepithelial carcinoma'/de OR 'intraepithelial carcinomas' OR 'intraepithelial neoplasm' OR 'intraepithelial neoplasms' OR 'intraepithelial neoplasia'/de OR 'intraepithelial neoplasias' OR 'intraepithelial cancer' OR 'leukoplakia'/de OR leukoplakias OR leukoplakic OR 'keratosis'/de OR keratoses OR 'leukokeratosis'/de OR 'proliferative verrucous leukoplakia'/de OR 'erythroplasia'/de OR erythroplasias OR 'erythroplakia'/de OR 'erythroleukoplakia'/de OR 'oral submucous fibrosis'/de OR 'submucous fibrosis' OR 'oral lichen planus'/de OR 'cheilitis'/de OR 'actinic cheilosis' OR 'actinic cheilitis'/de OR 'actinic keratoses' OR 'actinic keratosis'/de OR 'reverse-cigar smokers' OR 'smokers palate' OR 'reverse smokers' OR 'oral lupus erythematosus' OR 'dyskeratosis congenita'/de OR 'oral lichenoid lesions' OR 'oral lichenoid lesion'/de OR 'oral graft versus host disease' OR 'oral graft-versus-host disease') AND ('mouth'/de OR mouths OR oral OR orals OR 'lip'/de OR 'lips'/de OR 'tongue'/de OR 'mouth mucosa'/de OR buccal OR 'palate'/de OR palates OR 'mouth floor'/de OR 'cheek mucosa'/de OR alveolar OR 'gingiva'/de OR gingivas OR 'gum'/de OR gums OR 'interdental papilla'/de OR 'interdental papillae'/de OR commissure OR 'maxillary tuberosity'/de OR 'uvula'/de OR uvular)	**3034**
**Web of Science**	(("Truth Disclosure" OR "Truth-telling" OR "Truth telling" OR "Telling the Truth" OR truth OR truthing OR truths OR "Access to Information" OR "Professional-Patient Relations" OR "Health Planning Guidelines" OR "Health Planning" OR guideline OR guidelines OR "Planning Guideline" OR "Planning Guidelines" OR "Guidelines for Health Planning" OR recommendations OR recommendation OR "Health Planning Recommendations" OR "Planning Recommendations" OR communication OR communications OR misinformation OR "Teach-Back Communication" OR "Closed-Loop Communication" OR "Closed Loop Communication" OR "Closed-Loop Communications" OR "Teach-Back" OR "Closed-Loop" OR	**1220**

	"Health Communication" OR "Health Communications" OR "Communication Barriers" OR "Communication Barrier" OR "Dentist-Patient Relations" OR "Dentist-Patient Relation" OR "Dentist Patient Relation" OR "Dentist-Patient Relationship" OR "Dentist Patient Relationship" OR "Dentist-Patient Relationships" OR "Dentist Patient Relationships" OR "Physician-Patient Relations" OR "Physician-Patient Relation" OR "Physician Patient Relation" OR "Physician Patient Relationship" OR "Physician Patient Relationships" OR "Physician Patient Relations" OR "Physician Patient Relations" OR "Doctor Patient Relations" OR "Doctor Patient Relation" OR "Doctor-Patient Relations" OR "Doctor-Patient Relation" OR "Delivering bad news" OR "bad news" OR "receiving bad news" OR "deliver bad news" OR "communicate bad news" OR "Communication of bad news" OR "Breaking bad news" OR "break bad news" OR protocol OR protocols OR "Clinical Protocols" OR "Clinical Protocol" OR questionnaires OR questionnaire OR "Quality of Life")) AND TEMA: (("Precancerous conditions" OR precancerous OR "pre-cancer" OR precancer OR preneoplastic OR "pre-neoplastic" OR "precursor lesion" OR "precursor lesions" OR "potentially malignant disorder" OR "potentially malignant disorders" OR "potentially malignant lesion" OR "potentially malignant lesions" OR premalignant OR "pre malignant" OR "pre malignancy" OR "pre malignancy" OR "pre malignancies" OR "pre malignancies" OR "Carcinoma in situ" OR "in situ carcinoma" OR "Carcinomas in situ" OR "in situ carcinomas" OR "preinvasive carcinoma" OR "preinvasive carcinomas" OR "pre-invasive carcinoma" OR "pre-invasive carcinomas" OR "intraepithelial carcinoma" OR "intraepithelial carcinomas" OR "intraepithelial neoplasm" OR "intraepithelial neoplasms" OR "intraepithelial neoplasia" OR "intraepithelial neoplasias" OR "intraepithelial cancer" OR leukoplakia OR leukoplakias OR leukoplakic OR keratosis OR keratoses OR leukokeratosis OR leukokeratosis OR "proliferative verrucous leukoplakia" OR erythroplasia OR erythroplasias OR erythroplakia OR erythroleukoplakia OR "Oral submucous fibrosis" OR "Submucous Fibrosis" OR "Oral Lichen Planus" OR cheilitis OR "actinic cheilosis" OR "Actinic cheilitis" OR "Actinic Keratoses" OR "Actinic Keratosis" OR "Reverse-cigar smokers" OR "smoker's palate" OR "reverse smokers" OR "Oral Lupus Erythematosus" OR "Dyskeratosis Congenita" OR "Oral Lichenoid Lesions" OR "Oral Lichenoid Lesion" OR "Oral graft versus host disease" OR "oral graft-versus-host disease")) AND TEMA: ((mouth OR mouths OR oral OR orals OR lip OR lips OR tongue OR "Mouth Mucosa" OR buccal OR palate OR palates OR "Mouth Floor" OR "cheek mucosa" OR alveolar OR gingiva OR gingivas OR gum OR gums OR "interdental papilla" OR "interdental papillae" OR commissure OR "maxillary tuberosity" OR uvula OR uvular))	
**Google Scholar**	(“Truth Disclosure" OR "Truth telling" OR "Professional-Patient Relations" OR Communication) AND (precancer OR "potentially malignant disorders” OR "pre malignancies") AND (Mouth OR "Oral cavity")	**100**

Supplementary Table S2. Excluded articles and the reasons for exclusion (*n*=13)

#	Author (year)	Reasons for Exclusion*	Reference
**1**	Crossan L & Conway DI (2019)	1	Crossan L, Conway DI. Another oral cancer clinical guideline - but does it propose changes to dental practice? Evid Based Dent. 2019; 20(1):7-8.
**2**	Brocklehurst *et al.* (2010)	3	Brocklehurst PR, Baker SR, Speight PM. A qualitative study examining the experience of primary care dentists in the detection and management of potentially malignant lesions. 1. Factors influencing detection and the decision to refer. Br Dent J. 2010; 208(2): E3; discussion 72-3.
**3**	Graner KM et al. (2016)	1	Graner KM, Rolim GS, Moraes ABA, Padovani CR, Lopes MA, Santos-Silva AR, Ramos-Cerqueira ATA. Feelings, perceptions, and expectations of patients during the process of oral cancer diagnosis. Support Care Cancer. 2016; 24(5):2323-2332.
**4**	Llewellyn CD et al. (2006)	1	Llewellyn CD, McGurk M, Weinman J. How satisfied are head and neck cancer (HNC) patients with the information they receive pre-treatment? Results from the satisfaction with cancer information profile (SCIP). Oral Oncol. 2006; 42(7):726-34.
**5**	Burkhart NW et al. (1997)	4	Burkhart NW, Burkes EJ, Burker EJ. Meeting the educational needs of patients with oral lichen planus. Gen Dent. 1997; 45(2):126-32.
**6**	Brocklehurst *et al.* (2010)	3	Brocklehurst PR, Baker SR, Speight PM. Primary care clinicians and the detection and referral of potentially malignant disorders in the mouth: a summary of the current evidence. Prim Dent Care. 2010; 17(2):65-71.
**7**	Sri Varsha et al. (2019)	4	Sri Varsha, L.; Krishnan, M. Quality of life in patients with precancerous lesions - A short review of literature. Drug Invention Today. 2019; 11: 2800-2803
**8**	Aghi, M. B (2014)	4	Aghi, M. B. Successful strategies of education and communication to prevent the occurrence of oral cancer among rural Indian population. Journal: Asia-Pacific Journal of Clinical Oncology. 2014; 10: 58.
**9**	Ogden G.R. (2010)	5	Ogden G.R. Summary of: A qualitative study examining the experience of primary care dentists in the detection and management of potentially malignant lesions. 2. Mechanics of the referral and patient communication. G. R. Ogden. British Dental Journal volume 208, pages74–75 (2010) Cite this article. Published: 23 January 2010.
**10**	Green, R et al. (2011)	4	Green, R.; Thomson, P. J.; Exley, C.; Steele, J. G. Understanding the transition from primary to secondary care: Experiences of patients with oral precancer. British Journal of Oral and Maxillofacial Surgery. 2011; 49: S60.
**11**	Chen SC et al. (2009)	1	Chen SC, Lai YH, Liao CT, Chang JT, Lin CC. Unmet information needs and preferences in newly diagnosed and surgically treated oral cavity cancer patients. Oral Oncol. 2009; 45(11): 946-52.
**12**	Satheeshkumar, et al. (2015)	4	Satheeshkumar, P. S.; Mohan, M. P. Unmet needs in oral precancerous patients in dental oncology department. Head and Neck. 2015; 37: E71.
**13**	Awojobi O et al. (2016)	1	Awojobi O, Newton JT, Scott SE. Pilot study to train dentists to communicate about oral cancer: the impact on dentists' self-reported behaviour, confidence and beliefs. Br Dent J. 2016; 220(2):71-6.

Legend: (1) studies of oral conditions other than OPMD; (2) potentially malignant conditions in anatomical sites other than the oral cavity; (3) clinical trials focused only on screening, risk factors, diagnosis or diagnostic test accuracy, and treatment of OPMD; (4) laboratory research with animal experimentation and *in vitro* studies, conference abstracts, posters, book chapters, and full-text not available; (5) overlapping information, we included the most recently reported or those providing more data.
